# Cross‐cultural differences in object recognition: Comparing asylum seekers from Sub‐Saharan Africa and a matched Western European control group

**DOI:** 10.1002/acp.3419

**Published:** 2018-05-29

**Authors:** Gabi de Bruïne, Annelies Vredeveldt, Peter J. van Koppen

**Affiliations:** ^1^ Department of Criminal Law and Criminology Vrije Universiteit Amsterdam Amsterdam The Netherlands

**Keywords:** asylum seekers, cross‐cultural psychology, eyewitness memory, object recognition, signal detection theory

## Abstract

Nowadays, more and more people report about their memories in cross‐cultural contexts. In international criminal settings and asylum procedures, object recognition tests can provide valuable information, for example, about weapons used during a crime or landmarks from the claimed region of origin. This study was the first to compare object recognition performance by asylum seekers from Sub‐Saharan Africa to a matched Western European control group. African participants performed worse than European participants on perceptual tests involving transformations from two‐ to three‐dimensional representations, but both groups performed equally well on an object recognition test that involved transformation from three‐ to two‐dimensional representations. However, African participants were significantly more likely to respond “yes” on the recognition test (i.e., an acquiescence response style) than European participants. Our findings elucidate cultural differences in responding on an object recognition test. Judges, juries, and immigration officials would be wise to take these differences into account when evaluating recognition performance in cross‐cultural contexts.

## 1 INTRODUCTION

As a consequence of recent global developments, more and more individuals report about their memories in cross‐cultural contexts. People from Sub‐Saharan Africa are particularly likely to be questioned by investigators from a different cultural background. For example, the International Criminal Court in The Hague, the Netherlands, is currently investigating 10 situations, of which 8 took place in Sub‐Saharan Africa (International Criminal Court, n.d.). Combs (2010, 2017) and Anders (2011) provide a host of illustrative examples of cultural challenges that have arisen in criminal cases about conflicts in Rwanda, Sierra Leone, and Liberia. Some African witnesses, particularly from rural areas, find it difficult to date crimes they have witnessed and judge the elapse of time in hours, days, or months, because they do not routinely keep track of dates and times. In international criminal cases, dates and times are often crucial because the suspect may have a solid alibi for one time but not for another. In a similar vein, witnesses are sometimes unable to estimate other properties, such as distances, the size of objects, or the number of people during an incident (Anders, 2011; Combs, 2010). That presents challenges for fact‐finding in legal cases, because judges need to know exactly where and how a crime took place. One crucial question, for example, may be what kind of weapon was used by the perpetrator. To our knowledge, this study is the first to experimentally examine differences in object recognition between Sub‐Saharan African participants and a matched Western control group.

Another context in which the recognition of objects can play a crucial role is in immigration interviews. An asylum seeker may be asked to identify buildings or objects from the region from which he/she claims to originate (cf. Van Veldhuizen et al., 2017; Van Veldhuizen, Horselenberg, Landström, Granhag, & Van Koppen, 2017). These types of questions are asked because immigration officials think that they can judge the validity of asylum seekers' statements by evaluating their answers. Putting aside for the moment the more general misconception that people can deduce whether someone is telling the truth based on their story (see e.g., Bond & DePaulo, 2006; Vredeveldt, Van Koppen, & Granhag, 2014), an additional problem in cross‐cultural contexts is that evaluators' expectations of what a statement should look like is based on their own cultural background (Herlihy, Jobson, & Turner, 2012). This is problematic because most immigration officials hail from Western societies, which typically have an individualistic culture (Triandis, 1989), whereas asylum seekers often come from African societies (Eurostat, 2017), which typically have a collectivistic culture (Triandis, 1989). Cross‐cultural psychological research shows that people from collectivistic cultures do not report about events in the same way as people from individualistic cultures do (e.g., Jobson, 2009; Wang, 2001).

Cultural differences are not limited to the way in which people report about the events, they also extend to the perception of objects. As early as the start of the 20th century, Rivers (1901, 1905) showed that people from India and the Torres‐Strait Islands were more susceptible to one visual illusion (the horizontal–vertical illusion), 1The horizontal–vertical illusion consists of a horizontal line and a vertical line of the same length, arranged in the shape of an inverted T. Observers typically overestimate the length of the vertical line relative to the horizontal line. but less susceptible to another visual illusion (the Müller‐Lyer illusion), 2The Müller‐Lyer illusion consists of two horizontal lines, one with arrowheads on both ends pointing inwards and one with arrowheads on both ends pointing outwards. Observers typically overestimate the length of the horizontal line with the arrowheads pointing outwards. than English participants. Subsequent research replicated these findings with African groups. For example, researchers found that Koisan people from rural South Africa were not susceptible to the Müller‐Lyer illusion (Segall, Campbell, & Herskovits, 1966) and South African participants from rural areas were less susceptible to the rotating‐trapezoid‐window illusion than South African participants from urban areas (Allport & Pettigrew, 1957). 3The rotating‐trapezoid‐window illusion is a window that appears to be rectangular but is, in fact, a trapezoid. The window is mounted on a rod connected to an electric motor that rotates it continuously around its vertical axis. It is typically misperceived to be oscillating, reversing its direction once every 180°. Extension of these findings with a depth perception test developed by Hudson (1960) showed that participants from rural traditional societies in South Africa (Hudson, 1960), Ghana (Jahoda & McGurk, 1974; Mundy‐Castle, 1966), and Uganda (Kilbride & Robbins, 1969) were less likely to perceive depth in pictures than participants from comparable urban samples. The most commonly heard explanation for these differences in visual perception is that people from urban societies are exposed more frequently to rectangular shapes, writing, illustrations, and photographs than people from African rural societies. Visual illusions such as the Müller‐Lyer illusion and the rotating‐trapezoid‐window illusion rely on the viewer's familiarity with rectangular shapes, buildings, and windows (e.g., Allport & Pettigrew, 1957). Similarly, depth perception based on pictures requires acceptance of graphical conventions commonly encountered in illustrations and photographs (e.g., Hudson, 1960).

Most existing literature addresses the transformation of two‐dimensional (2D) into three‐dimensional (3D) representations, even though the other way around (3D to 2D) seems to be more relevant in judicial contexts. After all, witnesses typically view an event in real life (3D) and are subsequently asked to recognize objects (e.g., weapons) or people (e.g., perpetrators) based on photographs (2D). These recognition tests may take the form of a show‐up, in which the witness is asked whether the photograph presented to them depicts the person or object they saw during the crime, or a line‐up, in which the witness is asked to make a selection from a series of photographs. In the same vein, in order to verify claims in immigration interviews, asylum seekers may be asked to identify a landmark that they saw in real life (3D), such as the church from their hometown, from a photograph, or a series of photographs (2D). To our knowledge, this study is the first to examine cultural differences in the transformation of 3D into 2D representations.

In this study, we assessed whether individuals from Sub‐Saharan African countries differ from a matched Western European control group in their recognition of common objects. Specifically, we tested how well both groups were able to recognize African‐style and European‐style vases. We predicted that participants would be better at recognizing vases from their own continent (cf. Bovet & Vauclair, 2000). Additionally, we manipulated whether the object recognition test was 3D (vases placed in front of participant) or 2D (vases shown on photographs). Based on findings that Sub‐Saharan African participants tend to have more difficulty transforming 2D to 3D representations than Western European participants (e.g., Jahoda & McGurk, 1974), we hypothesized that the same would be true for the reverse ability, in this case, transforming the encoded 3D vases into 2D representations. We therefore expected that Sub‐Saharan African participants would perform worse on the 2D recognition test than Western European participants. To enable comparisons with previous studies in which 2D to 3D transformations were assessed, we also included a depth perception test and a visuospatial processing test to examine this ability. Again, we predicted that Sub‐Saharan African participants would have more difficulty with these tests.

In addition to basic differences between participant groups in object recognition, depth perception, and visuospatial processing, we also explored potential explanations for differences in performance. In previous work (e.g., Allport & Pettigrew, 1957; Hudson, 1960), it has been suggested that African participants are less likely to perceive depth in pictures and succumb to visual illusions than Western participants because they are exposed less frequently to rectangular shapes and 2D representations, for example, because they live in rural areas and do not have access to newspapers, magazines, and TV. We therefore asked the Sub‐Saharan African participants in our sample whether they had come from a rural or urban environment, whether they had owned a TV or computer screen in their home country, and for how long they had been in Europe at the time of testing. We predicted that African participants from rural backgrounds, those with minimal exposure to screens and those who had only recently arrived in Europe, would perform worse on the depth perception, visuospatial processing, and 2D recognition tests compared with African participants from urban backgrounds, and those with more exposure to screens.

## 2 METHOD

### 2.1 Participants

Forty‐six Sub‐Saharan African participants (30 male) were recruited from one of four asylum seeker centres in the Netherlands. They had come from Eritrea (*N* = 21), Sudan (*N* = 7), Nigeria (*N* = 6), Ethiopia (*N* = 3), Somalia (*N* = 3), Democratic Republic of the Congo (*N* = 2), Burundi (*N* = 1), Rwanda (*N* = 1), Togo (*N* = 1), and Uganda (*N* = 1). For the sake of brevity, we will refer to them as “African” from this point forward. The African participants had been in the Netherlands for 12.50 months on average (*SD* = 14.81; range: 1–96 months). Half of them indicated that they originated from a village (*N* = 23) and half from a city (*N* = 23). They were between 18 and 46 years old (*M* = 30.30; *SD* = 7.62). The highest level of education they had completed varied from none (*N* = 5), primary school (*N* = 10), high school (*N* = 17), higher vocational education (*N* = 3), to university (*N* = 11). Finally, 34 African participants indicated that they had owned at least one screen (TV or computer) in their home country; 12 indicated that they had not owned a TV or computer.

Western European control participants were selected based on their match to the African participants in our sample in terms of gender, rural/urban background, age, and educational level. For the sake of brevity, we will refer to them as “European” from this point forward. Forty‐six European participants (27 male) were recruited via the researchers' personal networks at different locations in the Netherlands. All of them were from the Netherlands; just over half from a village (*N* = 24) and the other half from a city (*N* = 22). European participants' ages ranged from 19 to 54 (*M* = 31.33; *SD* = 10.14). The highest level of education they had completed varied from primary school (*N* = 2), high school (*N* = 18), higher vocational education (*N* = 9), to university (*N* = 17).

Our sample size of 46 participants per participant group allowed us to detect medium‐sized effects of *d* = 0.60 with power = .80 at the standard .05 alpha error probability. As a result of the matching procedure, there were no significant differences between the two groups in terms of gender distribution, χ^2^(1) = 1.13, *p* = .395, Cramer's *V* = .11; rural/urban background, χ^2^(1) = 0.04, *p* = .999, Cramer's *V* = .02; or participant age, *U* = 1022.50, *p* = .784, *η* ^*2*^ = .00. We also tried to match on educational level, but it proved impossible to find European participants with levels of education comparable with the African group. The frequencies reported above show that European participants had a significantly higher level of education than African participants in our sample, χ^2^(4) = 14.65, *p* = .004, Cramer's *V* = .40. This difference will be addressed in the analyses and Section 4.

### 2.2 Materials

#### 2.2.1 Depth perception

**Figure 1:**
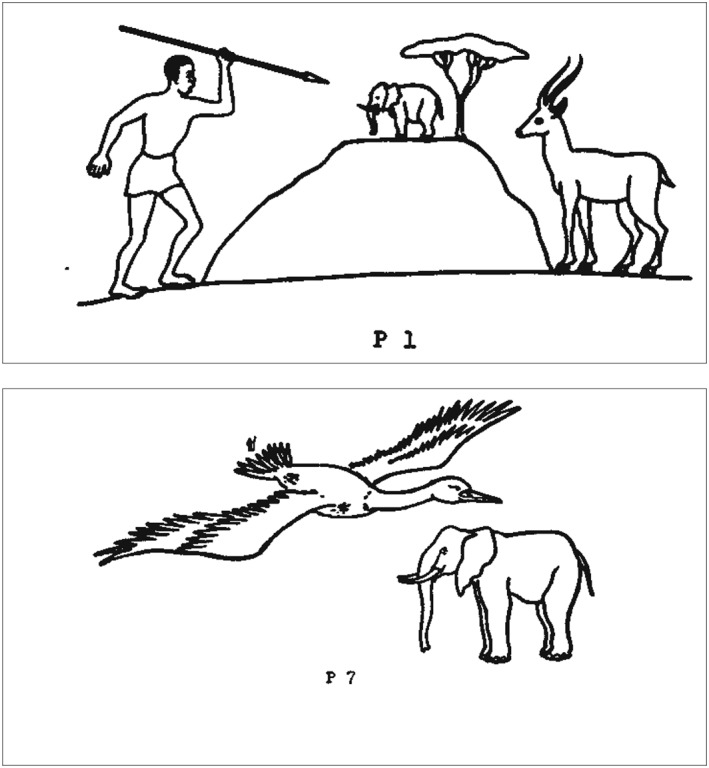
Drawing 1 (top) and 7 (bottom) of the Hudson Pictorial Depth Perception Test (Hudson, 1960). The man in the bottom drawing is depicted small, just above the bird's tail

To assess depth perception, we used an adapted version of the Hudson Pictorial Depth Perception Test (Hudson, 1960). This test consists of 11 drawings and 1 photograph—we used only the drawings because the photograph is of poor quality. Drawings 1–6 measure depth perception in the horizontal dimension (i.e., scanning an image from the left to the right, or vice versa). All six drawings depict a hunting scene in which a man aims a spear at an elephant and an antelope (see Figure 1). The elephant is always in‐between the man and the antelope and is depicted as smaller than the antelope. In Drawings 2 and 3, some of the elements in the illustration overlap. In Drawings 4, 5, and 6, perspective is added in the form of a road that disappears across the horizon. For each drawing, participants were asked three questions: (a) “What do you see?,” (b) “What is the man doing?,” and (c) “Which animal is closer to the man?.” The responses to Questions b and c indicate whether participants perceive depth in the drawings: if they respond that the man is hunting the antelope and that the antelope is closer to the man than the elephant, those responses are coded as 3D.

Drawings 7–11 measure depth perception in the vertical dimension (i.e., scanning an image from the top to bottom, or vice versa). All five drawings depict a flying bird, an elephant, and a man (see Figure 1). The bird is always depicted as larger than the elephant, and the elephant is always larger than the man. In Drawings 8 and 9, some of the elements in the illustration overlap, and in Drawings 10 and 11, perspective is added in the form of a road. For each drawing, participants were asked two questions: (a) “What do you see?” and (b) “Which animal is closer to the man?.” The response to Question b indicates whether participants perceive depth in the drawings: if they respond that the elephant is closer to the man than the bird, that response is coded as 3D.

For each 3D response provided on the depth perception test, the participant scored one point. Participants could score two points per drawing for the horizontal dimension (Drawings 1–6) and one point per drawing for the vertical dimension (Drawings 7–11), for a total maximum score of 17 points on the depth perception test.

#### 2.2.2 Visuospatial processing

**Figure 2:**
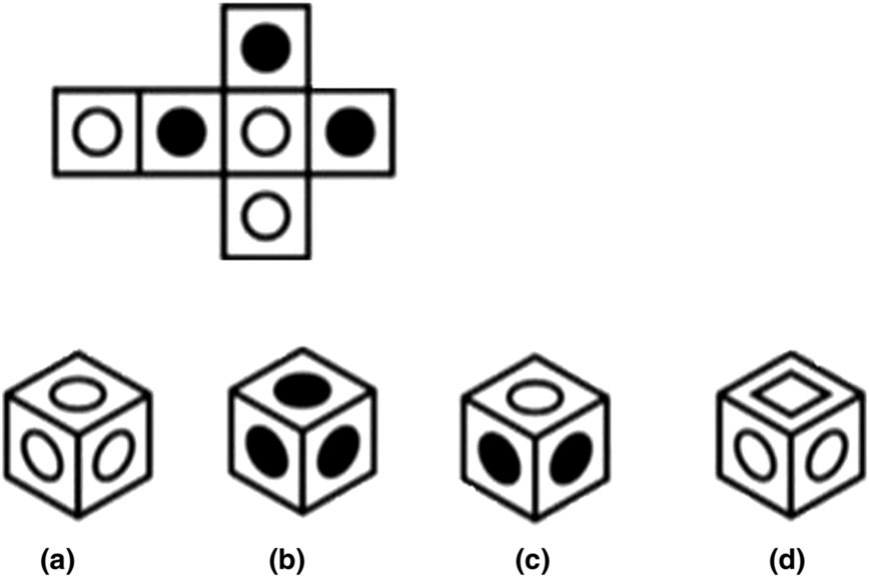
Example item from the Differential Aptitude Test: Space Relations (Bennett et al., 1973)

For the visuospatial test, we used four items from the Differential Aptitude Test: Space Relations (Bennett, Seashore, & Wesman, 1973). The items are four‐alternative forced‐choice questions that measure the ability to move from a 2D to a 3D representation. The participant is shown a 2D pattern of a cube and is instructed to mentally fold the pattern to create the cube (see Figure 2). The participant needs to select from four alternatives which cube can be created from the pattern. Participants scored one point per correct response, for a total maximum score of four points on the visuospatial test.

#### 2.2.3 Object recognition

**Figure 3:**
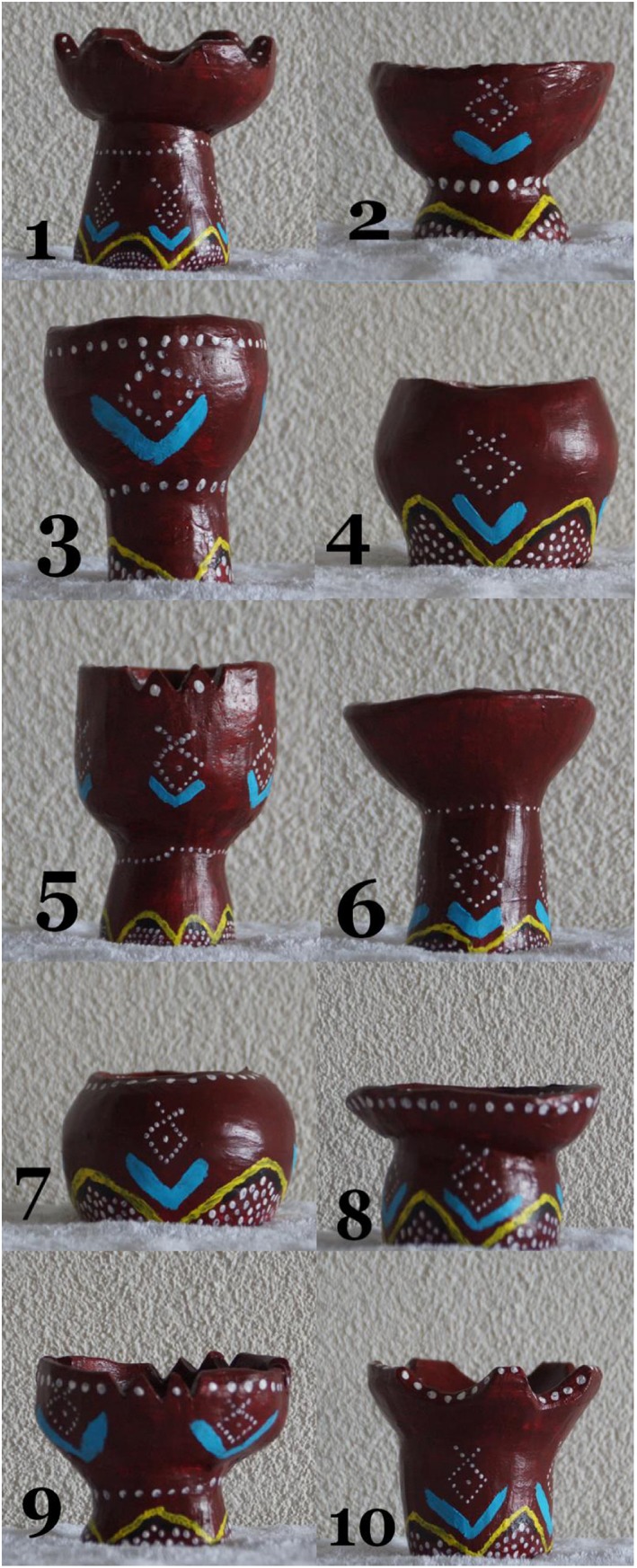
The 10 African vases in the recognition test [Colour figure can be viewed at http://wileyonlinelibrary.com]

**Figure 4:**
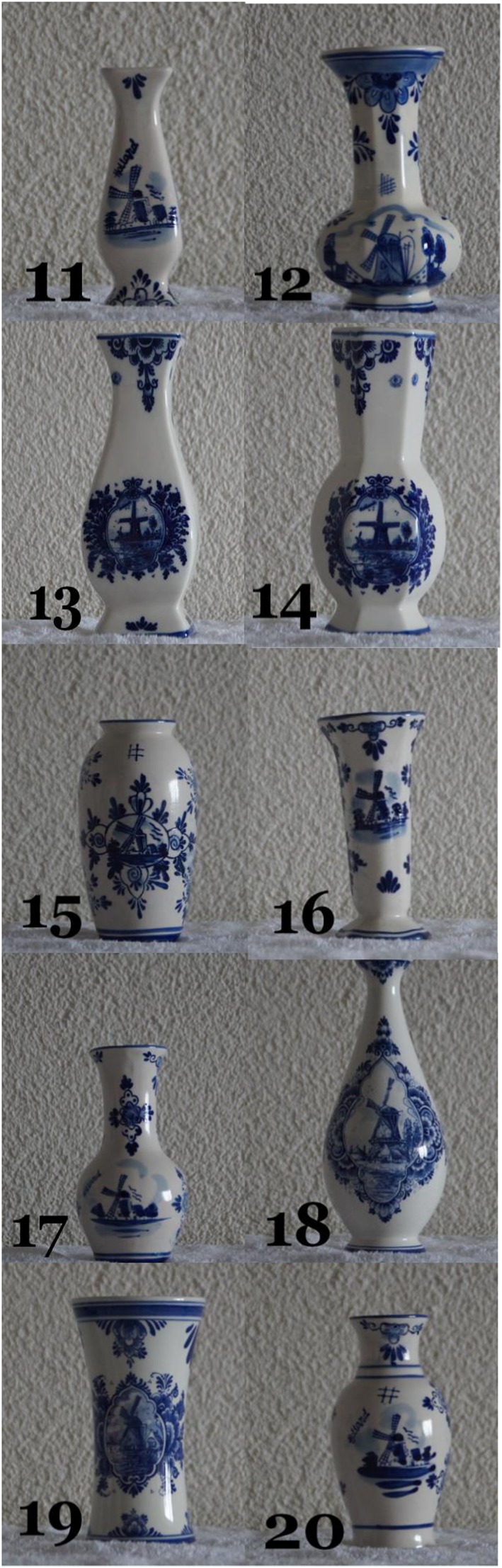
The 10 Dutch vases in the recognition test [Colour figure can be viewed at http://wileyonlinelibrary.com]

Twenty vases served as stimulus materials in the recognition test. We sculpted, baked, and painted 10 African vases, which were typical Eritrean incense holders (see Figure 3), and we bought 10 European vases, which were typical Dutch Delft Blue pottery (see Figure 4). All vases within each category had different shapes, but were painted in a similar fashion.

### 2.3 Procedure

The data from African participants were collected across a period of 8 weeks and the matched European control participants were tested in the 6 weeks that followed. Each participant was tested individually. At each experimental location, two rooms were used: one for the encoding phase and another for the perceptual tests and recognition phase. The research with European participants was conducted in Dutch. When possible, the research with African participants was conducted in English. For participants whose English language proficiency was insufficient (*N* = 16), a telephone‐based interpreter service was used. These were all professional interpreters employed by the Central Agency for the Reception of Asylum Seekers (COA) and other government agencies.

At the start of the session, participants were informed that their participation was completely voluntary and that they could end their participation at any time. Participants were not compensated for their time. After providing informed consent, participants were told that they would go into another room, where they would see 10 vases on a table. In the other room, they were shown five African and five European vases. All participants viewed the same 10 vases, placed together in the same configuration on a table. They were instructed to look at the vases carefully. Participants were able to see the vases from all angles. They were allowed to view the vases for 90 s in total, after which they left the room.

In the next room, participants first provided demographic information (e.g., age, gender, time in Europe, educational background, urban/rural background, and exposure to screens) and then completed the visuospatial and depth perception tests. Approximately 15 min after they had encoded the vases, participants were presented with a recognition test. Participants were informed that they would see 20 vases, one by one. All 20 vases (10 old and 10 new) were presented sequentially in random order. Participants in each group were randomly assigned to one of two viewing conditions: the vases were either placed before the participant on a table (3D condition) or shown on photographs (2D condition). For each vase, participants were asked to indicate if they had seen it on the table earlier that day (yes/no).

## 3 RESULTS

**Figure 5:**
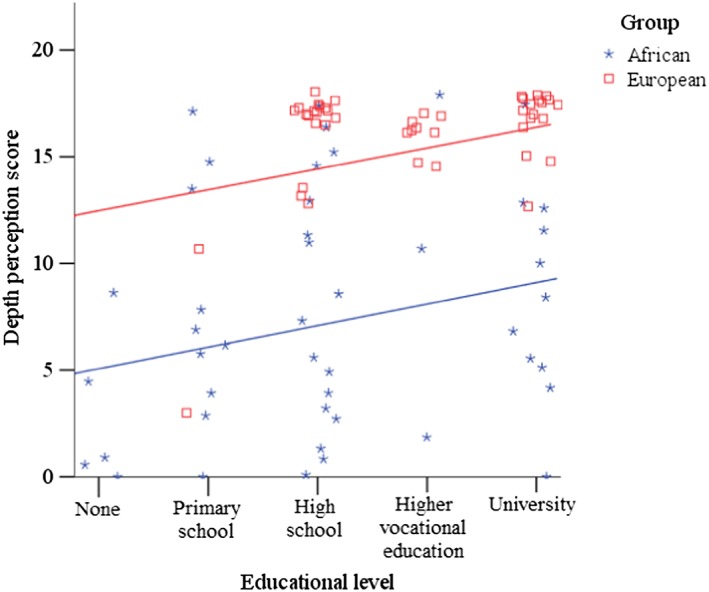
Scores on the depth perception test as a function of educational level, displayed separately for each participant group. Fit lines reflect linear regression slopes (African participants: *R* ^*2*^ = .06, European participants: *R* ^*2*^ = .15) [Colour figure can be viewed at http://wileyonlinelibrary.com]

To account for the fact that we were unable to match participant groups on education, we entered educational level as a covariate in all analyses. 4Note that the assumption of independence between the covariate and the independent variable only applies to independent variables that are directly manipulated by the experimenter (Grace‐Martin, 2012). Because Participant Background (African, European) was not, and cannot be, directly manipulated, the fact that it was related to Educational Level is irrelevant for the analysis of covariance. Crucially, there was independence between Educational Level and the independent variable that was directly manipulated: Recognition Format. Visual inspection of scatterplots for each dependent variable (see Figure 5 for an example) revealed that the data patterns for the group of African participants with low levels of education (none or primary school)—which could not be matched to European participants because nearly all of them had higher levels of education—did not diverge from the data patterns for the other participants, rendering the data suitable for analysis of covariance (ANCOVA). Prior to the analyses, we checked all relevant assumptions. When assumptions of normality or homogeneity of variance were not met, we double‐checked the parametric findings with nonparametric tests and provide medians in addition to means and standard deviations. All reported *p* values are two‐tailed.

### 3.1 Depth perception

Scores on the depth perception test ranged from 0 to 17. Because the data violated assumptions of normality (significant negative skew: *z* = 3.01) and homogeneity of variance, *F*(1, 90) = 37.74, *p* < .001, we checked the findings with nonparametric tests, which confirmed all parametric results. To examine differences in depth perception scores between African and European participants, we conducted an ANCOVA with educational level as a covariate. We found a significant effect of educational level, *F*(1, 89) = 6.91, *p* = .010, *η* ^*2*^ = .07. Unsurprisingly, participants with a higher level of education performed better on the depth perception test (see Figure 5). After controlling for educational level, there was still a significant and large difference between African participants (*M* = 7.57, *SD* = 5.51, *Mdn* = 6.50) and European participants (*M* = 15.89, *SD* = 2.49, *Mdn* = 17.00), *F*(1, 89) = 68.31, *p* < .001, *η* ^*2*^ = .43.

Whereas Europeans performed close to ceiling, Africans showed much greater variance in performance (see Figure 5). We explored three potential explanations for this variance: urban/rural background, exposure to TV/computer screens in their home country, and time in Europe. African participants from a city performed marginally better (*M* = 9.00, *SD* = 5.40, *Mdn* = 9.00) than African participants from a village (*M* = 6.13, *SD* = 5.35, *Mdn* = 6.00), *t*(44) = 1.81, *p* = .077, *d* = 0.53, 95% CI [−0.06, 1.12]. Similarly, African participants who had owned a screen in their home country performed marginally better (*M* = 8.38, *SD* = 5.31, *Mdn* = 8.00) than African participants who had not owned a screen (*M* = 5.25, *SD* = 5.63, *Mdn* = 4.00), *t*(44) = 1.73, *p* = .091, *d* = 0.57, 95% CI [−0.09, 1.25]. Neither of these trends was statistically significant though, possibly due to a lack of statistical power (e.g., only 12 African participants had not owned a screen in their home country). Finally, a simple linear regression revealed that time in Europe did not significantly predict performance on the depth perception test, *R* ^*2*^ = .05, *F*(1, 43) = 2.50, *p* = .121. 5The data for time in Europe contained one outlier that was more than two standard deviations higher than the mean: a participant who had been in Europe for 96 months (Z = 5.64). That participant was removed from all analyses involving time in Europe.


### 3.2 Visuospatial processing

Scores on the visuospatial test ranged from 0 to 4. The data violated assumptions of normality (significant platykurtosis: *z* = 2.80) and homogeneity of variance, *F*(1, 90) = 5.29, *p* = .024, but nonparametric tests confirmed all parametric results reported below. An ANCOVA with educational level as a covariate revealed a significant effect of education, *F*(1, 89) = 12.31, *p* < .001, *η* ^*2*^ = .12. Participants with a higher level of education performed better on the test (no education: *Mdn* = 0, primary school: *Mdn* = 1, high school: *Mdn* = 2, higher vocational and university education: *Mdn* = 4). After controlling for educational level, there was still a significant and large difference between groups, *F*(1, 88) = 34.55, *p* < .001, *η* ^*2*^ = .28. European participants achieved significantly higher scores (*M* = 3.30, *SD* = 0.94, *Mdn* = 4.00) than African participants (*M* = 1.63, *SD* = 1.32, *Mdn* = 1.00).

Again, European participants performed close to ceiling, whereas African participants showed much greater variance in performance on the test. We therefore explored potential explanatory variables. We found no significant difference between African participants originating from a city (*M* = 1.83, *SD* = 1.53, *Mdn* = 1.00) versus a village (*M* = 1.43, *SD* = 1.08, *Mdn* = 1.00), *t*(39.60) = 1.00, *p* = .321, *d* = 0.29, 95% CI [−0.29, 0.88]. Similarly, we found no significant difference between African participants who had owned a screen (*M* = 1.79, *SD* = 1.39, *Mdn* = 1.00) versus not owned a screen (*M* = 1.17, *SD* = 1.03, *Mdn* = 1.00) in their home country, *t*(44) = 1.43, *p* = .160, *d* = 0.47, 95% CI [−0.19, 1.14]. It should be noted again that nonsignificant results may be due to low power. Finally, a simple linear regression revealed that time in Europe did not significantly predict visuospatial processing performance, *R* ^*2*^ = .04, *F*(1, 43) = 1.64, *p* = .208.

### 3.3 Object recognition accuracy

**Figure 6:**
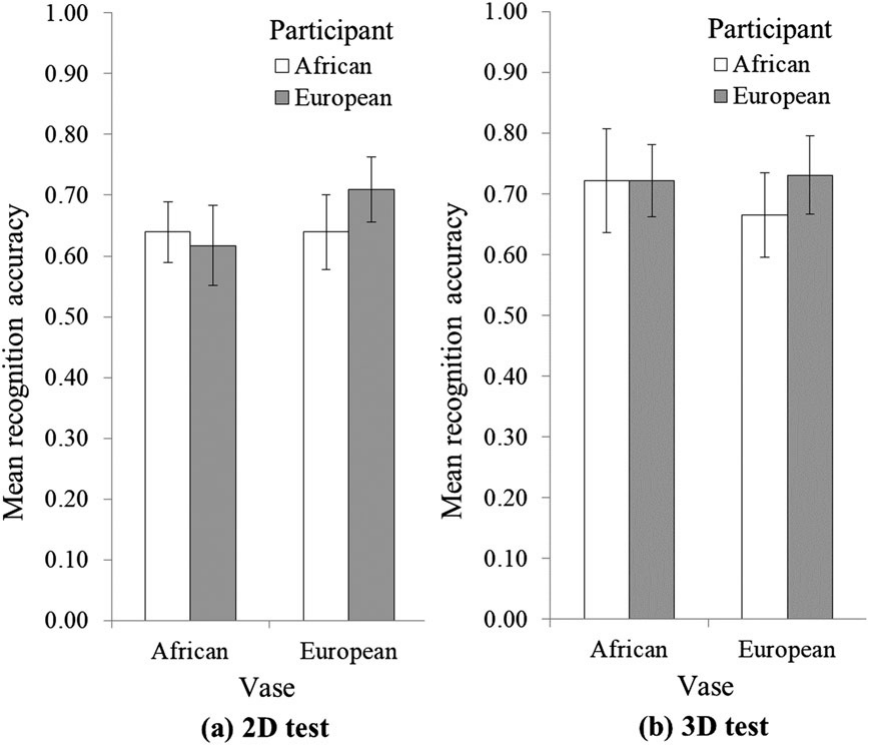
African and European participants' recognition accuracy for African and European vases on (a) the 2D test and (b) the 3D test

The proportion of correct responses on the object recognition test (i.e., overall recognition accuracy; see Figure 6) was subjected to a 2 (Participant Background: African, European) × 2 (Recognition Format: 2D, 3D) × 2 (Type of Vase: African, European) mixed ANCOVA with type of vase as a within‐participant factor and Educational Level as a covariate. There was a significant effect of Educational Level, *F*(1, 87) = 4.48, *p* = .037, *η* ^*2*^ = .05: participants with some form of higher education achieved higher recognition accuracy than participants who had not completed any higher education (no education: *M* = .66, *SD* = .13; primary school: *M* = .63, *SD* = .10; high school: *M* = .64, *SD* = .13; higher vocational education: *M* = .72, *SD* = .07; university: *M* = .72, *SD* = .11). The ANCOVA revealed no significant main effects of Participant Background, *F*(1, 87) = 0.24, *p* = .628, *η* ^*2*^ = .00, or Type of Vase, *F*(1, 87) = 0.41, *p* = .526, *η* ^*2*^ = .00, but there was a significant effect of Recognition Format, *F*(1, 87) = 5.64, *p* = .020, *η* ^*2*^ = .06. Participants in the 3D condition achieved significantly higher recognition accuracy (*M* = .70, *SD* = .13) than participants in the 2D condition (*M* = .65, *SD* = .09).

Our prediction that African participants would have more difficulty with the 2D recognition test than European participants was not confirmed: the interaction between Participant Background and Recognition Format was not significant, *F*(1, 87) = .04, *p* = .848, *η* ^*2*^ = .00 (see Figure 6). Our prediction that participants would be better at recognizing vases from their own continent was not confirmed either: the interaction between Participant Background and Type of Vase was not significant, *F*(1, 87) = 2.13, *p* = .148, *η* ^*2*^ = .02. None of the other interactions were significant (all *F*s < 3.04, all *p*s > .086).

Within the African sample, we explored potential explanatory variables for differences in performance. We found no significant difference between African participants originating from a city (*M* = .65, *SD* = .14) versus a village (*M* = .66, *SD* = .12), *t*(44) = −0.11, *p* = .911, *d* = −0.03, 95% CI [−0.61, 0.55]. However, African participants who had owned a screen in their home country achieved significantly higher recognition accuracy (*M* = .69, *SD* = .13) than those who had not owned a screen (*M* = .57, *SD* = .08), *t*(44) = 3.05, *p* = .004, *d* = 1.01, 95% CI [0.33, 1.71]. It should again be noted that interpretation of these findings is limited by the fact that our sample included only 12 participants who had not owned a screen in their home country. Finally, a simple linear regression revealed that time in Europe did not significantly predict recognition accuracy, *R* ^*2*^ = .00, *F*(1, 43) = 0.14, *p* = .708.

### 3.4 Signal detection analysis

To assess the extent to which the recognition accuracy data were driven by true discrimination between old and new vases, as opposed to a tendency to respond liberally or conservatively on the recognition test, we conducted signal detection analysis. Correct‐positive and false‐positive responses on the object recognition test were used to calculate discrimination accuracy (*d′*) and response criterion (*c*). Prior to calculation, proportions of 0 and 1 were converted to 1/(2 N) = .05 and 1–1/(2 N) = .95, respectively (MacMillan & Creelman, 1991).

Across the total sample, *d′* ranged from −0.51 to 2.93, with higher positive values indicating better discrimination accuracy. A 2 × 2 × 2 mixed ANCOVA on *d′* revealed a significant effect of Educational Level, *F*(1, 88) = 4.93, *p* = .029, *η* ^*2*^ = .05. Participants with some form of higher education tended to discriminate better between old and new vases (no education: *M* = 0.95, *SD* = 0.81; primary school: *M* = 0.79, *SD* = 0.66; high school: *M* = 0.83, *SD* = 0.77; higher vocational education: *M* = 1.22, *SD* = 0.44; university: *M* = 1.28, *SD* = 0.70). The ANCOVA revealed no significant main effects of Participant Background, *F*(1, 87) = 0.37, *p* = .547, *η* ^*2*^ = .00, or Type of Vase, *F*(1, 87) = 0.40, *p* = .841, *η* ^*2*^ = .00, but a marginally significant effect of Recognition Format, *F*(1, 87) = 3.85, *p* = .053, *η* ^*2*^ = .04. Participants tended to achieve higher discrimination accuracy on the 3D test (*M* = 1.17, *SD* = 0.82) than on the 2D test (*M* = 0.87, *SD* = 0.57). There were no significant interactions (all *F*s < 2.13, all *p*s > .148).

Across the total sample, response criterion *c* ranged from −1.61 (more liberal) to 0.64 (more conservative). Because the data violated the assumption of homogeneity of variance, *F*(1, 90) = 11.47, *p* = .001, we checked the findings with nonparametric tests. 6The nonparametric tests confirmed all parametric results except one: a Wilcoxon signed‐rank test showed that participants responded significantly more liberally to African vases (*M* = −0.31, *SD* = 0.56, *Mdn* = −0.29) than to European vases (*M* = −0.11, *SD* = 0.56, *Mdn* = 0.00), *T* = 1011.00, *p* < .001. A 2 × 2 × 2 mixed ANCOVA on *c* revealed no significant effect of Educational Level, *F*(1, 87) = 0.04, *p* = .847, *η* ^*2*^ = .00. There was also no significant effect of Type of Vase, *F*(1, 87) = 0.21, *p* = .649, *η* ^*2*^ = .00, but there were significant effects of both Participant Background, *F*(1, 87) = 16.42, *p* < .001, *η* ^*2*^ = .16, and Recognition Format, *F*(1, 87) = 6.05, *p* = .016, *η* ^*2*^ = .06. African participants were significantly more likely to respond liberally (i.e., say “yes”) on the recognition test (*M* = −.039, *SD* = 0.55, *Mdn* = −0.40) than European participants (*M* = −0.01, *SD* = 0.29, *Mdn* = 0.00). Further, participants were significantly more likely to respond liberally on the 2D recognition test (*M* = −0.31, *SD* = 0.46, *Mdn* = −0.21) than on the 3D test (*M* = −0.10, *SD* = 0.47, *Mdn* = 0.00). There were no significant interactions (all *F*s < 1.45, all *p*s > .232).

## 4 DISCUSSION

We examined differences between Sub‐Saharan asylum seekers and a matched Western European control group in their performance on a depth perception test, a visuospatial processing test, and an object recognition test. After controlling for differences in educational level between the two groups, we still found significant differences between African and European participants in their performance on the depth perception and visuospatial processing tests. In line with previous findings, European participants achieved higher scores on those tests than African participants. In contrast, we found no significant difference between groups in accuracy on the object recognition test: after controlling for educational level (which was positively associated with recognition accuracy), African participants performed just as well as European participants. Interestingly, however, African participants were significantly more likely to respond “yes” on the recognition test than European participants. Each of these findings will be discussed in turn.

Our findings on the depth perception and visuospatial processing tests replicated previous findings that people from Sub‐Saharan Africa have more difficulty transforming a 2D representation into a 3D representation (Hudson, 1960; Jahoda & McGurk, 1974; Kilbride & Robbins, 1969; Mundy‐Castle, 1966). These findings have previously been explained by the idea that people from traditional African societies have less exposure to illustrations and photographs depicting 3D objects and scenes than people from modern Western societies (e.g., Hudson, 1960). To explore this potential explanation, we asked African participants whether they originated from a rural or urban background, whether they had owned a TV or computer screen in their home country, and for how long they had been in Europe. None of these variables were associated with significant differences in performance on the visuospatial processing test. African participants who had come from a village and who had not owned a screen in their home country scored a little, but nonsignificantly, lower on perceiving depth in the pictures than African participants who had come from a city and who had owned a screen. That hints to the idea that more exposure to rectangular buildings and TV or computer screens may be associated with an increased ability to transform 2D representations to 3D representations. The interpretation of these findings is limited, however, by the fact that our sample included only 12 African participants who had not owned a screen in their home country. It is further limited by the fact that most African participants in our sample were probably exposed to many screens during the time they had spent in asylum seeker centres located in urban areas in the Netherlands (a year on average). Thus, further research involving participants with more limited exposure to 2D representations is required to make a cleaner comparison.

In contrast, African participants did not have more difficulty than European participants in transforming 3D representations (vases placed in front of them during the encoding phase) to 2D representations (photographs of vases presented in the recognition phase) on the object recognition test. African participants did show poorer performance on the 2D recognition test than on the 3D recognition test, but so did European participants. The lack of differences in accuracy between groups suggest that the ability to transform a 3D representation into a 2D representation may tap into a different cognitive skill than the ability to transform a 2D representation into a 3D representation. Whereas previous research has focused on the latter ability, the former is much more likely to be relevant in judicial contexts. Eyewitnesses encode an event (3D) and are subsequently asked to recognize people or objects from photographs (2D), not the other way around. Similarly, asylum seekers may be asked to recognize landmarks they have seen in real life (3D) based on photographs presented to them (2D), not the other way around. This study represents the first step in examining cultural variations in the ability to recognize objects that have been viewed in real life based on photographic representations. More research on this ability to move from 3D to 2D could prove valuable in informing legal decisions about recognition performance in cross‐cultural contexts.

Based on Bovet and Vauclair's (2000) findings that familiarity with stimulus objects improves recognition performance, we predicted that participants would recognize vases from their own continent better than vases from a different continent. We found no support for this prediction. In hindsight, our familiarity manipulation may have been too weak. After all, the African participants had been in the Netherlands for a year on average already, and would likely have encountered the typical Delfts Blue pottery style in that period. Similarly, the European participants in our sample would likely have encountered the African‐style vases before, for example, on holiday, on TV programmes about Africa, or even in Dutch homes, in which “exotic” decorations such as these are not uncommon.

There were no significant differences between African and European participants in overall recognition accuracy or discrimination accuracy on the object recognition test. Thus, African participants were just as likely as European participants to correctly recognize a previously seen object. Interestingly, however, we did find a significant difference in response criterion. African participants were much more likely to respond that they had seen a presented object before, regardless of whether they had actually seen it. In other words, African participants were more likely to say “yes” to the experimenter. This tendency is known as the acquiescence response style (see e.g., Cronbach, 1942; Martin, 1964). Cultural differences in acquiescence response style have similarly been observed in previous studies (for an overview, see Cheung & Rensvold, 2000). According to Cheung and Rensvold, they may be explained by differences in social desirability, beliefs about the value of high scores, or concerns about one's own ability. The African participants in our sample likely originated from collectivistic cultures (Triandis, 1989), in which the desire to agree with a conversational partner—particularly with an authority figure, such an experimenter—may outweigh the desire to provide an accurate response (see e.g., Markus & Kitayama, 1991). In this study, saying “yes” did not indicate agreement with the conversational partner, because the experimenter did not express an opinion. Nevertheless, a general habit to say “yes” in daily life on the part of the African participants could explain their tendency to say “yes” in this experiment as well. In the individualistic cultures typical of Western European societies, on the other hand, less value is placed on agreeableness. This might explain why African participants displayed an acquiescence response style, whereas European participants did not.

One limitation of this study was that we were unable to match participant groups on education. To remedy this problem, we included educational level as a covariate in all analyses. Although education did have an impact on performance, with more highly educated participants achieving higher accuracy on all tests, we still found significant differences between participant groups even after controlling for educational level. This means that the difference in educational level between African and European participants is not sufficient to explain the observed differences in performance. Future research should strive to compare different cultural groups that are comparable in educational level, to disentangle effects of education and cultural background. From a practical perspective, however, it should be noted that these factors are entangled in real life as well. On average, an individual from Sub‐Saharan Africa will have received less education than a gender‐, age‐, and background‐matched individual from Western Europe. Therefore, Western European judges' and immigration officials' expectations of what a statement should look like may not only be coloured by their own cultural background (Herlihy et al., 2012), but also by their own educational level.

If the current findings extend to the recognition of other types of objects (e.g., weapons) and persons, this would have important implications in judicial contexts. Although our African participants did not differ in recognition accuracy from European participants, they were significantly more likely to say “yes”. When a suspect is shown in a fair line‐up, this is not a major problem: The witness may be more likely to pick someone from the line‐up, but if he chooses randomly, he is much more likely to pick a filler than to pick the suspect (for more on the fairness of line‐ups, see e.g., Malpass, Tredoux, & McQuiston‐Surrett, 2007). The erroneous selection of a filler is not associated with negative consequences for the selected person, because all fillers are known to be innocent, and will thus not be prosecuted. Oftentimes, however, the suspect is shown in an unfair line‐up, in which the fillers are not real options for the witness (e.g., because they do not resemble the perpetrator) or the suspect stands out in some way (e.g., because he is wearing different clothes than the fillers). This means that even someone who has not witnessed the crime can easily point out which of the members in the line‐up is the police suspect. Or even worse, the suspect may be shown without any alternative options at all. In a show‐up procedure, the witness views a photograph of the suspect and is asked whether this is the perpetrator. In unfair line‐ups and show‐ups, an increased tendency to say “yes” is a major problem, because the witness is highly likely to falsely identify the suspect. We know from exoneration data that false identifications are the greatest contributing factor to wrongful convictions (Innocence Project, n.d.; Gross & Shaffer, 2012). Of course, the present findings do not speak directly to person identifications in legal settings. If the acquiescence response style observed in this study extends to person identifications, however, this would mean that unfair line‐ups and show‐ups may be particularly problematic when used with Sub‐Saharan African witnesses.

In conclusion, it is clear from current and previous findings that cognitive processes that were traditionally thought to be universal do in fact differ between cultural groups. Yet, the vast majority of psychological research has involved participants from a Western, Educated, Industrialized, Rich, and Democratic background (Henrich, Heine, & Norenzayan, 2010). Findings from previous studies on eyewitness memory, person identification, and object recognition may therefore not generalize to people from different cultures. This study replicated previous findings showing that African participants performed worse than European participants on perceptual tests involving transformations from 2D to 3D representations. For an object recognition test that required transformation from 3D to 2D representations, accuracy rates were comparable across groups, but African participants were significantly more likely to respond “yes” (i.e., an acquiescence response style) than European participants. Police officers, judges, juries, and immigration officials would be wise to take cultural variations into account in their evaluations of statements made by individuals from a different culture.
